# Defective Glyoxalase 1 Contributes to Pathogenic Inflammation in Cystic Fibrosis

**DOI:** 10.3390/vaccines9111311

**Published:** 2021-11-11

**Authors:** Marilena Pariano, Claudio Costantini, Ilaria Santarelli, Matteo Puccetti, Stefano Giovagnoli, Vincenzo N. Talesa, Luigina Romani, Cinzia Antognelli

**Affiliations:** 1 Department of Medicine and Surgery, University of Perugia, 06132 Perugia, Italy; marilena.pariano@gmail.com (M.P.); claudio.costantini@unipg.it (C.C.); ilasanta@libero.it (I.S.); vincenzo.talesa@unipg.it (V.N.T.); luigina.romani@unipg.it (L.R.); 2 Department of Pharmaceutical Science, University of Perugia, 06132 Perugia, Italy; matteo.puccetti@gmail.com (M.P.); stefano.giovagnoli@unipg.it (S.G.)

**Keywords:** glyoxalase 1, cystic fibrosis, anakinra

## Abstract

Cystic fibrosis (CF) is an autosomal recessive disorder that affects multiple organs, although a decline in respiratory function represents the major cause of morbidity and mortality. The airways of CF patients are characterized by a chronic inflammatory state to which the receptor for advanced glycation end-products greatly contributes. Glyoxalase 1 (GLO1) is the major enzyme metabolizing methylglyoxal, a potent precursor of advanced glycation end-products. Its role in CF has never been investigated. We herein resorted to murine and human preclinical models of CF to define the contribution of GLO1 to inflammatory pathology. We found that the expression and activity of GLO1, measured by real-time PCR and Western blot or a specific spectrophotometric assay, respectively, are defective in mice and human bronchial cells from CF patients exposed to *Aspergillus fumigatus*, a common pathogen in CF, but could be restored upon blockade of interleukin-1 receptor signaling by anakinra in mice. This study suggests that GLO1 contributes to pathology in CF and may be potentially targeted to mitigate inflammation.

## 1. Introduction

Cystic fibrosis (CF) is the most common autosomal recessive disorder in Caucasians. It is caused by mutations in the gene encoding for the chloride/bicarbonate channel cystic fibrosis transmembrane conductance regulator (CFTR), and more than 2000 variants have been reported to date that may variously affect protein expression, maturation, and function [1]. Although CF is a multi-organ disease, the major cause of morbidity and mortality is represented by the progressive decline of respiratory functions [1]. A dysfunctional CFTR results in increased production and viscosity of mucus, with several pathogenic consequences. Mucus plugging generates hypoxia and death of epithelial cells by necrosis, with the release of damage-associated molecular patterns (DAMPs) and sterile neutrophilic inflammation. Moreover, mucociliary clearance is impaired with increased microbial colonization and infection. The defective resolution phase of the inflammatory response finally results in a chronic inflammatory state that characterizes the airways of CF patients [2]. Therefore, a defective CFTR function, an increased susceptibility to infection, and the hyperinflammatory state all contribute to the respiratory decline of CF patients, by playing independent or interconnected roles [2]. In this regard, we have previously demonstrated a molecular mechanism linking the inflammatory response induced by hypoxia to that resulting from microbial infection [3]. Specifically, hypoxia up-regulated the receptor for advanced glycation end-products (RAGE) that is activated by the advanced glycation end-products (AGEs) formed by non-enzymatic reaction between reducing sugars and proteins, lipids, and nucleic acids. However, RAGE also binds other ligands, which are either endogenous, such as the DAMPs high mobility group 1 and members of the S100 family, or exogenous, such as PAMPs and dietary AGEs, and has been implicated in several chronic inflammatory diseases [4], including CF [5,6]. Indeed, treatment with soluble RAGE, an inhibitor decoy receptor, was able to prevent fungal growth and reduce inflammatory pathology [3], thus highlighting the importance of RAGE in driving inflammation in CF by coupling host- and microbial-derived stimuli.

Methylglyoxal (MG) is a highly reactive dicarbonyl compound mainly generated as a side product of glycolysis by non-enzymatic phosphate elimination from glyceraldehyde phosphate and dihydroxyacetone phosphate [7,8]. MG is recognized as a major precursor of AGEs mainly by reacting with the free amino group of arginine residues [8]. Since MG accumulation is cytotoxic, an efficient detoxification pathway exists that is based on the glyoxalase system, consisting of two enzymes, i.e., the rate-limiting enzyme GLO1, GLO2, and reduced glutathione (GSH) [8]. A dysfunctional glyoxalase system has been associated with several pathological conditions [9], with disparate mechanisms. Indeed, increased glyoxalase activity has been identified as an evasion mechanism employed by tumor cells to prevent cell death. Conversely, reduced glyoxalase activity, with accumulation of MG, has been observed in aging, diabetes, cardiovascular disease, and other chronic inflammatory conditions. Although CF is characterized by a chronic inflammatory state at least in part driven by RAGE activation, it is unknown whether altered MG levels and glyoxalase activity are present in CF and could be potentially targeted as an anti-inflammatory strategy.

Based on these premises, in the present study, we have evaluated the expression and functional activity of GLO1 in murine models of CF and human bronchial epithelial cells from CF patients upon challenge with *Aspergillus fumigatus* conidia and found that GLO1 was defective in both murine and human models. We could also demonstrate that anakinra, the recombinant version of the interleukin-1 receptor antagonist, that has been shown to reduce inflammatory pathology in murine and human CF [10,11,12], was able to rescue GLO1 in CF, thus indicating that GLO1 represents a potential target to dampen inflammation in CF.

## 2. Materials and Methods

### 2.1. Mice, Infections, and Treatments

Eight- to ten-week-old male and female C57BL6 wild-type mice were purchased from Jackson Laboratories (Bar Harbor, Maine). CF mice homozygous for the Phe508del-Cftr allele, which had been backcrossed for 12 generations to the C57BL/6 strain (Cftr^tm1EUR^, abbreviated *Cftr^F508del^*), were obtained from B. Scholte (Erasmus Medical Center) [13]. These mice were provided with a special food consisting of an equal mixture of SRM-A (Arie Blok, Woerden, the Netherlands) and Teklad 2019 (Harlan Laboratories, Indianapolis, IN, United States), and water acidified to pH 2.0 with HCl and containing 60 g/L PEG 3350, 1.46 g/L NaCl, 0.745 g/L KCl, 1.68 g/L NaHCO_3_, and 5.68 g/L Na_2_SO_4_. Newborn mice were genotyped by cutting a small piece of tail 12 d after birth. Four mice per group were used in each experiment. Mice were anesthetized in a plastic cage by inhalation of 3% isoflurane (Forane, Abbott, Chicago, IL, United States) in oxygen before intranasal instillation of 2 × 10^7^ *A. fumigatus* (Af293) resting conidia per 20 μL of saline. Mice were sacrificed by cervical dislocation at 6 days postinfection (dpi). Quantification of fungal growth was performed as described [14]. Briefly, fungal growth was expressed as colony-forming units (log10 CFU), obtained by serially diluting homogenates on Sabouraud agar plates incubated at 35 °C for 24 h. Bronchoalveolar lavage (BAL) was performed by cannulating the trachea and washing the airways with 3 × 0.5 mL of PBS to collect the BAL fluid. For differential BAL fluid cell counts, cytospin preparations were made and stained with May–Grünwald Giemsa reagents (Sigma–Aldrich, St. Louis, MO, United States). For histology, lungs were removed and immediately fixed in 10% neutral buffered formalin (Bio-Optica Milano Spa, Milan, Italy) for 24 h. The fixed organs were dehydrated, embedded in paraffin, sectioned into 3–4 μm, and stained with Periodic Acid-Schiff reagent (Abcam). Mice were treated intraperitoneally with 10 mg/kg anakinra reconstituted in sterile water daily for 6 consecutive days beginning the day of the infection, a dose that mimics human therapeutic dosages and is pharmacologically active in mice [10,14,15,16,17] Mice were sacrificed a day after treatment. Infections were performed under isoflurane anesthesia, and all efforts were made to minimize suffering. Mouse experiments were performed according to Italian Approved Animal Welfare Authorization 360/2015-PR and Legislative Decree 26/2014, regarding the animal license obtained by the Italian Ministry of Health lasting for 5 years (2015–2020).

### 2.2. Cells and Treatments

Stable lentiviral-based transductions of the parental CFBE41o- cells, a CF human bronchial epithelial cell line, derived from a CF patient homozygous for the ΔF508 CFTR mutation and immortalized with the origin-of-replication defective SV40 plasmid (pSVori-), with either WT CFTR or p.Phe508del-CFTR, were provided by L.J. Galietta. The transduced CFBE41o- cells were maintained in minimum Eagle’s medium supplemented with 50 U/mL penicillin, 50 μg/mL streptomycin, 2 mM l-glutamine, 10% FBS, and 1 μg/mL blasticidin (WT CFTR) or 2 μg/mL puromycin (p.Phe508del-CFTR) in a 5% CO_2_ and 95% air incubator at 37 °C. Cells were exposed or not to *A. fumigatus* conidia at a 1:20 cell/conidia ratio and concomitantly treated or not with 50 or 250 µM MG (Sigma–Aldrich, St. Louis, MO, USA).

### 2.3. Real-Time PCR

Real-time RT-PCR was performed in lung homogenates or CFBE41o- cells using the iCycler iQ detection system (Bio-Rad, Hercules, CA, United States) and SYBR Green chemistry (Finnzymes Oy, Espoo, Finland). Samples were lysed and total RNA was extracted using RNeasy Mini Kit (QIAGEN, Hilden, Germany) and was reverse transcribed with Sensiscript Reverse Transcriptase (QIAGEN, Hilden, Germany) according to the manufacturer’s directions. The mouse primers (5′–3′) were as follows: *Ager*: GGAATTGTCGATGAGGGGAC and CAACAGCTGATTGCCCTCTG; *Glo1*: TGTGCCTTTGAGGATTAATGGA and TCACCAAGTGCCCATAGCTG; *Hif1a*: TCAAGTCAGCAACGTGGAAG and TTCACAAATCAGCACCAAGC; *Il17a*: GACTACCTCAACCGTTCCAC and CCTCCGCATTGACACAGC; *Il1b*: TGACGGACCCCAAAAGATGAAGG and CCACGGGAAAGACACAGGTAGC; *Tnfa*: CGAGTGACAAGCCTGTAGCC and AAGAGAACCTGGGAGTAGACAAG. The human primers (5′–3′) were as follows: *Ager*: GCCAGAAGGTGGAGCAGTAG and CCAGTGGATTTGAGGAGAGG; *Glo1*: CGAGGGTCTGAATTGCCATTG and CTCTCCAGAAAAGCTACACTTGAG. Amplification efficiencies were validated and normalized against GAPDH. The thermal profile for SYBR Green real-time PCR was at 95 °C for 3 min, followed by 40 cycles of denaturation for 30 s at 95 °C and an annealing/extension step of 30 s at 60 °C. Each data point was examined for integrity by analysis of the amplification plot. The mRNA-normalized data were expressed as relative cytokine mRNA in treated vs. unstimulated samples.

### 2.4. Western Blot Analysis

Extraction of total proteins was performed by lysing 1.5 × 10^6^ cells in precooled radioimmunoprecipitation assay (RIPA) lysis buffer [18]. Protein concentration in cell extracts was determined spectrophotometrically using the BCA protein assay kit (Pierce, Waltham, MA, USA). Blots of cell lysates were incubated with antibodies against GLO1 (Santa Cruz Biotechnology, Dallas, TX, USA). Normalization was performed with β-actin antibody (Sigma–Aldrich, St. Louis, MO, USA). Chemiluminescence detection was performed with LiteAblotPlus chemiluminescence substrate (Euroclone) using the ChemiDoc XRS+ Imaging system (Bio-Rad), and quantification was obtained by densitometry image analysis using Image Lab 5.1 software (Bio-Rad, Hercules, CA, USA). Uncropped gels are shown in Figure S1.

### 2.5. GLO1 Enzymatic Activity

Extract preparation for enzymatic activity assays was performed as previously described [18]. GLO1 enzyme activity was assayed in lung homogenates by an established method [18]. Briefly, the assay solution contained 0.1 mol/L sodium phosphate buffer, pH 7.2, 2 mmol/L MG, and 1 mmol/L reduced glutathione (GSH). The reaction was monitored spectrophotometrically by following the increase in absorbance at 240 nm and 25 °C, attributable to the formation of S-d-lactoylglutathione. One unit of activity was defined as 1 µmol of S-d-lactoylglutathione produced per minute.

### 2.6. MG-H1 Detection

MG-H1 was measured using the OxiSelectTM methylglyoxal competitive enzyme-linked immunosorbent assay (ELISA) kit (Cell Biolabs, cat. # STA-011), according to the manufacturer’s instructions. 

### 2.7. Statistical Analysis

GraphPad Prism software 6.01 (GraphPad Software) was used for the analysis. Data are expressed as mean ± SEM. Statistical significance was calculated by one- or two-way ANOVA (Tukey’s or Bonferroni’s post hoc test) for multiple comparisons and by a two-tailed Student’s *t*-test for single comparison. The distribution of levels tested by a Kolmogorov–Smirnov normality test turned out to be non-significant. The variance was similar in the groups being compared. We considered all *p* values ≤ 0.05 significant.

## 3. Results

### 3.1. GLO1 Expression and Activity Are Defective in CF Mice with Aspergillosis

**Figure 1:**
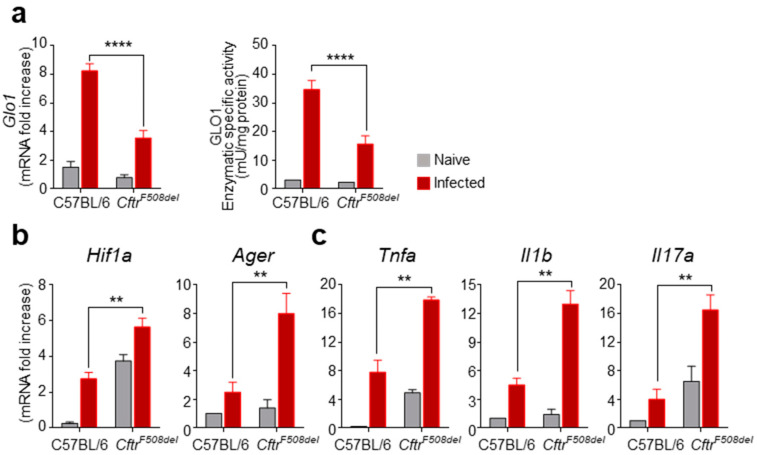
GLO1 expression and activity in CF mice. C57BL/6 and *Cftr^F508del^* mice were infected intranasally with live *A. fumigatus* conidia and evaluated for the levels of (**a**) GLO1 mRNA and enzyme specific activity, and for the levels of (**b**) *Hif1a*, *Ager*, and (**c**) *Tnfa*, *Il1b*, *Il17a* in the lung after 6 days of infection. Gene expression was performed by RT-PCR and enzyme-specific activity by an enzymatic assay as described in Section 2. Data are presented as mean ± SD of three independent experiments. Each in vivo experiment includes four mice per group. ** *p* < 0.01, **** *p* < 0.0001, infected C57BL/6 vs. infected Cftr^F508del^.

In order to assess whether the glyoxalase system was operative in CF, we evaluated the expression and activity of the rate-limiting enzyme GLO1 in the lungs of mice harboring the most common deletion of phenylalanine in position 508 (*Cftr^F508del^*) after intranasal infection with *A. fumigatus* conidia. We found a strong increase in GLO1 mRNA levels and enzymatic-specific activity in wild-type mice infected with *A. fumigatus* (Figure 1a) as opposed to the modest modulation of GLO1 expression and activity in *Cftr^F508de^*^l^ mice (Figure 1a), suggesting that GLO1 might be impaired in CF and unable to cope with the dicarbonyl stress expected in the hypoxic and inflammatory environment associated with the infection. In fact, we previously demonstrated, and here confirmed (Figure 1), that hypoxia promotes inflammation via RAGE in CF [3]. In particular, we found that the lungs of *Cftr^F508del^* mice had increased mRNA levels of *Hif1α*, both basally and after infection with conidia, compared to wild-type mice (Figure 1b). Moreover, the hypoxic environment elicited an increased expression of *Ager* encoding for RAGE (Figure 1b) and of the inflammatory cytokines *Tnfα*, *Il1β* and *Il17a* (Figure 1c). Thus, the hypoxic and inflammatory environment in CF may favor the accumulation of reactive dicarbonyls that are poorly scavenged to prevent cytotoxic damage. Together, these data indicate that the pro-inflammatory hypoxia–RAGE axis is paralleled by a decrease in GLO1 mRNA expression and activity in CF.

### 3.2. GLO1 Is Down-Regulated in Human CF

**Figure 2:**
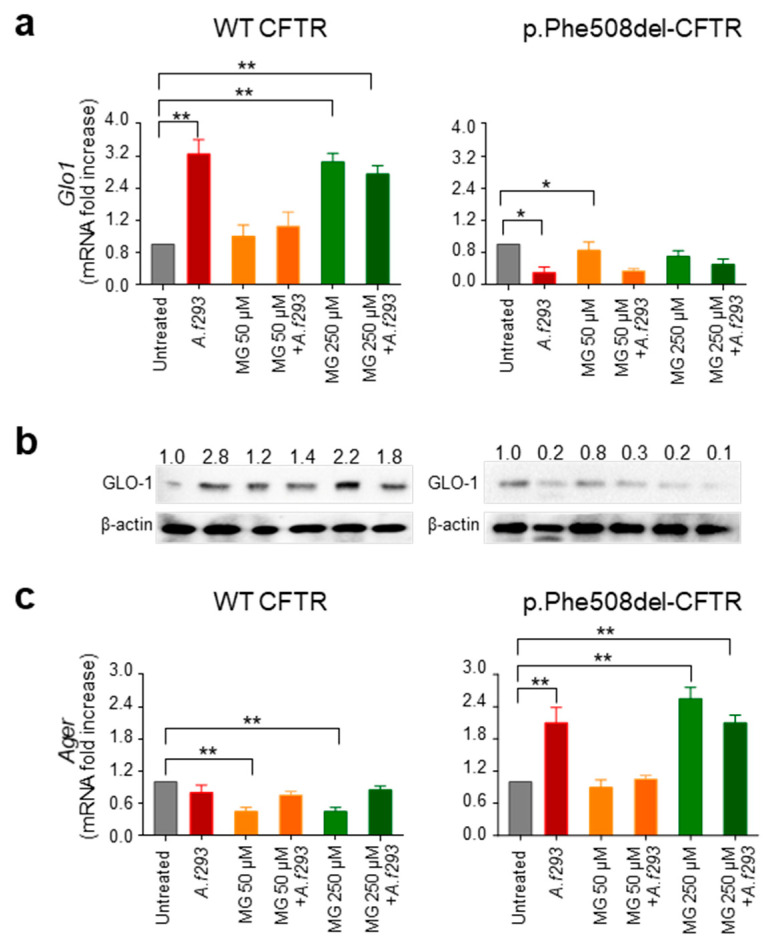
GLO1 is down-regulated in CF bronchial cells exposed to *A. fumigatus* conidia. CFBE41o- cells transduced with either wild-type or p.Phe508del-CFTR were treated with *A. fumigatus* conidia and/or methylglyoxal (MG) for 8 h and evaluated for (**a**) GLO1 mRNA and (**b**) protein levels, and (**c**) *Ager* mRNA levels. Protein levels were determined by Western blot analysis, and gene expression by RT-PCR. In panel b, numbers indicate intensity ratio. Data are presented as mean ± SD of three independent experiments. * *p* < 0.05, ** *p* < 0.01, treated vs. untreated.

To extend these findings in a human setting, we resorted to the human CF bronchial epithelial cell line CFBE41o- cells transduced with either wild-type or p.Phe508del-CFTR and challenged with *Aspergillus* conidia. As shown in Figure 2, GLO1 mRNA (a) and protein (b) expression were increased in CFBE41o- cells transduced with wild-type CFTR upon exposure to *Aspergillus* conidia. Accordingly, the expression of *Ager* encoding for RAGE was not increased by conidia (Figure 2c), suggesting that the increased levels of GLO1 prevented AGEs from accumulating, thus keeping RAGE at low levels. On the contrary, challenge with *Aspergillus* conidia of CFBE41o- cells transduced with p.Phe508del-CFTR did not result in increased GLO1 mRNA and protein expression (Figure 2a,b), which were actually decreased, while the expression of *Ager* was up-regulated (Figure 2c). The same results were observed if CFBE41o- cells transduced with either wild-type or p.Phe508del-CFTR were treated with MG rather than conidia. These results would indicate that it is the accumulation of AGEs per se, rather than the specific stimulus, that triggers an opposite response in cells expressing wild-type or p.Phe508del-CFTR. In agreement with this conclusion, the challenge with conidia did not result in an additional effect when used in combination with MG, indicating that the effects of conidia on GLO1 and RAGE are mediated by accumulation of reactive dicarbonyls.

### 3.3. Anakinra Rescues GLO1 in Murine CF

**Figure 3:**
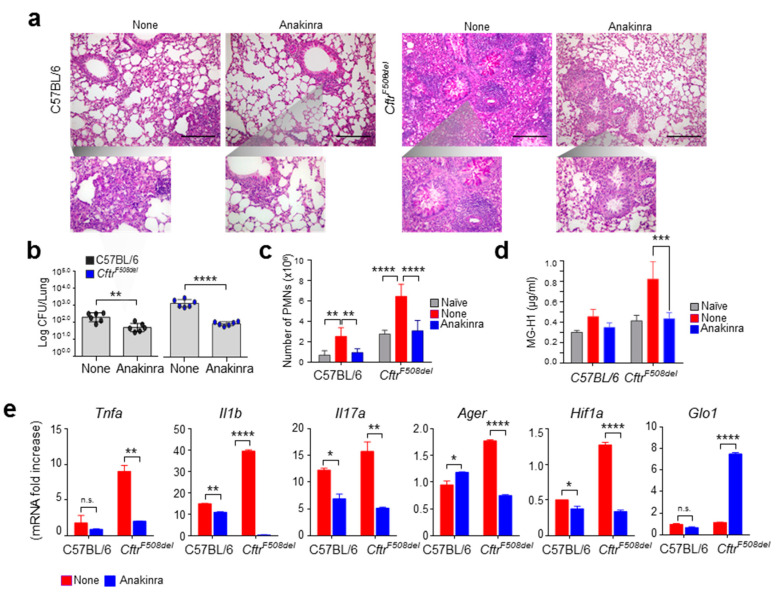
Anakinra rescues GLO1 in CF mice. C57BL/6 and Cftr^F508del^ mice were infected intranasally with live *A. fumigatus* conidia, treated with anakinra, and evaluated for (**a**) lung histopathology, (**b**) fungal growth (log10 cfu mean ± SEM), (**c**), neutrophil infiltration, (**d**) MG-H1 levels, and (**e**) *Tnfa*, *Il1b*, *Il17a*, *Ager*, *Hif1a* and *Glo1* expression at 6 dpi. Gene expression was performed by RT-PCR (data are presented as mean ± SD of three independent experiments). Images were taken with a high-resolution microscope (Olympus BX51), 20× and 40× magnification (scale bars, 200–100 μm). For histology, data are representative of three independent experiments. Each in vivo experiment includes four mice per group. * *p* < 0.05, ** *p* < 0.01, *** *p* < 0.001, **** *p* < 0.0001, Anakinra vs. None. n.s.: not significant.

It has previously been shown that anakinra, the recombinant form of IL-1 receptor antagonist, reduces inflammation in CF by down-regulating the pathogenic activity of the NLRP3 inflammasome [10] and reducing the hypoxia-driven inflammation [19]. Of interest, it has also recently been shown that blocking the IL-1 receptor activity partially up-regulates GLO1 in anaplastic thyroid cancer cells [20]. This last finding suggests that the anti-inflammatory activity of anakinra in CF could encompass the regulation of GLO1. To directly prove this, we evaluated the expression of GLO1 in wild-type and *Cftr^F508del^* mice infected with *A. fumigatus* upon treatment with anakinra and found that anakinra was able to reduce the inflammatory pathology (Figure 3a), fungal burden (Figure 3b), and pulmonary neutrophil infiltration (Figure 3c) in *Cftr^F508del^* mice. Notably, the levels of the major AGE coming from MG adduction of arginine residues in proteins (MG-H1) were higher in *Cftr^F508del^* compared to wild-type mice upon infection, but restored to normal levels by anakinra treatment (Figure 3d). This was associated with reduced levels of inflammatory cytokine levels (Figure 3e) in *Cftr^F508del^* mice. Of great interest, anakinra reduced the mRNA levels of *Hif1α* and *Ager* while increasing the levels of *Glo1* (Figure 3e). Collectively, these data indicate that GLO1 could represent a therapeutic target in CF, such that increasing its activity might interfere with the pathogenic HIF1α–RAGE axis, and identify anakinra as a multifaceted anti-inflammatory molecule in CF.

## 4. Discussion

The results presented in this study integrate our previous findings on the role of RAGE in the chronic inflammatory state in CF. Indeed, we have previously shown that microbial infection increased the levels of S100B that directly activated RAGE and targeting RAGE reduced infection and dampened inflammation [3]. We have now added a new piece to the story by showing that the glyoxalase system is defective in murine and human CF, thereby causing an accumulation of MG and AGEs during infection that further sustains the activation of RAGE and the inflammatory pathology. In agreement with our previous study, preventing RAGE activation by restoring GLO1 activity with anakinra reduced fungal burden and the inflammatory pathology, further emphasizing the relevance of the RAGE pathway in CF. Given the shared inflammatory pathways [10], it is likely that what is observed in aspergillosis also applies to infection with *Pseudomonas aeruginosa*, which requires further investigation. The detoxification of MG requires a catalytic amount of GSH, which leads to the spontaneous formation of MG-GSH hemithioacetal, in turn isomerized by GLO1 in S-d-lactoylglutathione. It has long been known that CF patients present an extracellular deficit of GSH and a low GSH/GSSG ratio, which result from the deficient transport of glutathione mediated by CFTR [21]. Although this would imply increased intracellular levels of GSH, this is not the case. Indeed, the intracellular content of GSH is reduced in CF cells likely because of a reduced cysteine regeneration by γ-glutamyltransferase, located on the outer surface of the plasma membrane and active on extracellular GSH [22]. Therefore, a plausible explanation for the impaired glyoxalase system in CF could be traced back to the reduced intracellular levels of GSH, in turn resulting in the accumulation of MG [23], with increased formation of AGEs and activation of RAGE. While reduced levels of GSH may represent a mechanism for the impairment of the glyoxalase system in CF, our results also indicate that the same expression and activity of GLO1 are reduced. Interestingly, this reduction seems to be linked to the mutation of CFTR, as the same cell line expressing either the wild-type or the mutated form of CFTR has different levels of GLO1 in response to the same stimuli. The Glo1 gene contains various regulatory elements, including antioxidant-response element, and nuclear factor erythroid 2-related factor (Nrf2) is a regulator of Glo1 expression [8]. Interestingly, CF airway epithelia are characterized by a dysfunction of Nrf2, and F508del CFTR correctors are able to restore Nrf2 function [24]. Therefore, it may be speculated that the redox imbalance in CF epithelia, in part dependent on the decreased GSH levels, and the dysfunctional response by impaired Nrf2, all concur to the deficit of the glyoxalase system that we have observed in murine and human CF.

Consistent with the inhibitory activity of IL-1β on GLO1 activity [20], anakinra was able to restore the expression of GLO1 in CF mice. Although currently approved for the treatment of rheumatoid arthritis and cryopyrin-associated periodic syndrome, anakinra is effective against a variety of inflammatory conditions, including CF. Mechanistically, anakinra protected against inflammation in CF by preventing the unrestrained activation of the NLRP3 inflammasome, restoring the process of autophagy [10] and regulating mucin and pro-inflammatory mediators’ secretions [11,12]. More recently, anakinra was found to activate superoxide dismutase 2 to mitigate mitochondrial oxidative stress [17]. Thus, anakinra may be beneficial in CF by promoting a variety of protective responses, including the antioxidant response and the redox balance.

In conclusion, our results demonstrate that the glyoxalase system is deficient in murine and human CF and that the increased dicarbonyl stress by MG accumulation is at least in part responsible for the activation of RAGE and the inflammatory phenotype. Therefore, targeting the GLO1/MG/RAGE axis might represent a therapeutic strategy to mitigate the chronic inflammatory state in CF.

## Data Availability

The data that support the findings of this study are available from the corresponding author upon reasonable request.

## Supplementary Materials

The following are available online at https://www.mdpi.com/article/10.3390/vaccines9111311/s1, Figure S1: Whole blots reported in Figure 2b.
