# Supplementary material for: Defective Glyoxalase 1 Contributes to Pathogenic Inflammation in Cystic Fibrosis
Source: Vaccines (Basel). 2021 Nov 11;9(11):1311. doi: 10.3390/vaccines9111311 (PMC8625157; doi:10.3390/vaccines9111311)
Supplement: Supplementary file 1 [file vaccines-09-01311-s001.zip › vaccines-1411122-supplementary.pdf]

Supplementary Material: Defective glyoxalase 1 contributes to pathogenic inflammation in cystic fibrosis

Marilena Pariano, Claudio Costantini, Ilaria Santarelli, Matteo Puccetti, Stefano Giovagnoli, Vincenzo N. Talesa, Luigina Romani, Cinzia Antognelli

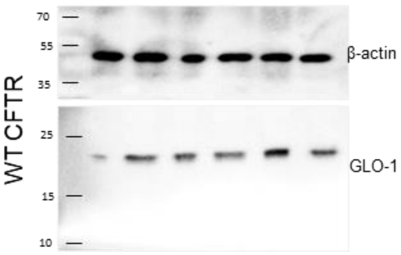

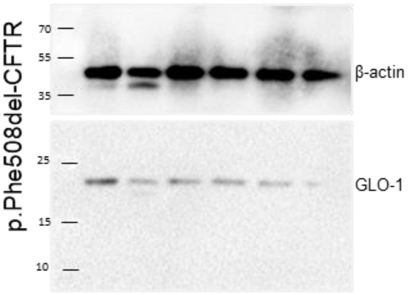

Figure S1. Whole blots reported in Figure 3.
